# Retraction: Evidence of brucellosis infection in the Milwaukee County Poor Farm Cemetery

**DOI:** 10.17912/micropub.biology.002176

**Published:** 2026-04-29

## Description

**Retraction notice:**

Title: Evidence of brucellosis infection in the Milwaukee County Poor Farm Cemetery

Authors: Helen Werner

microPublication Biology, 10.17912/micropub.biology.001601

The above article has been retracted at the request of the corresponding author, in agreement with the Editors. Documentation of institutional approval for the destructive testing conducted in this study was not recovered following a formal institutional records request. In the absence of recoverable documentation, retraction was carried out in the interest of transparency and research integrity.

Separately, inconsistencies were identified in the reporting of results across the abstract, figure, and methods section, which were not addressed because the article was retracted.

The microPublication Biology Editorial Team

